# Human Health; or the Influence of Atmosphere and Locality; Change of Air and Climate; Seasons; Food; Clothing; Bathing and Mineral Springs; Exercise; Sleep; Corporeal and Intellectual Pursuits, &c., on Healthy Man; Constituting Elements of Hygiene

**Published:** 1844-10

**Authors:** 


ART. VII.—*Human Health; or the Influence of Atmosphere and Locality; Change of Air and Climate; Seasons; Food; Clothing; Bathing and Mineral Springs; Exercise; Sleep; Corporeal and Intellectual Pursuits, &c., on healthy Man; Constituting Elements of Hygiene.* By ROBLEY DUNGLISON, M.D., Professor of the Institutes of Medicine, &c., in Jefferson Medical College of Philadelphia, &c., &c. A new Edition, with many Modifications and Additions. Philadelphia: Lea & Blanchard. 1844. 8vo. pp. 464.

It is seldom that we have retained a more lively recollection of the perusal of a professional work, than we still have of the pleasure with which we read the work before us, on its first appearance, several years ago. Dr. Rush tells us of a humorous old friend of his who insisted that his short memory was a blessing, because he could read a book a second time, after the lapse of a year or two, with as much pleasure as he enjoyed in the first perusal. We have to confess that it has not been so with us, in looking a second time through the pages of Dr. Dunglison's *Hygiene.* The impression made upon us by its instructive and pleasing details has lasted through a period in which many books have been forgotten; and the pleasure, therefore, of finding novelty in its pregnant facts and many curious narratives, is one which we must resign to others. We can most confidently recommend the volume to every class of readers; for who is not interested in the subjects of which it treats? The title-page sets these forth with such minuteness that we need not mention them.

The author, with his well known tact and industry, has brought his work up to the present state of the knowledge of the profession concerning hygiene. He thus speaks, in his preface, of the progress of the science, and of the modifications made in his treatise:

"Since the author published the first edition of his work, the subject of Hygiene has been more extensively and practically regarded, and, in many of its relations, has received the fostering care of governments. To this circumstance we are indebted for interesting sanitary inquiries, and valuable reports, and for various excellent contributions to vital statistics. These results of the researches of able investigators have modified materially many of the views which had been generally entertained in regard to the salubrity or insalubrity of different callings, and have led to a greater degree of attention on the part of philanthropists to the domestic condition of the poorer classes, to which rather than to their industrial relations, it would seem that many of the evils must be ascribed.

The author has subjected the first edition of his work to a complete revision, and has endeavored to notice, in this edition, the recent researches of distinguished hygienists. He has had a twofold object in view, as he had in preparing the first edition,—to produce a work which might serve the student as an accompaniment to the observations on Hygiene, that are made in Lectures on the Institutes of Medicine—and one which might equally enable the general reader to understand the nature of the action of various influences on human health, and assist him in adopting such means as may tend to its preservation."

Many of the topics embraced in this work open a wide field for controversy, and we see that some of the positions of the author have been already assailed by some of our contemporaries. We shall not engage in the discussion of any of these questions, for which much greater space than we have at our disposal would be necessary, but content ourselves with remarking that the views of Professor Dunglison, in the main, are sound and judicious, and that he has brought together, from all quarters, an amount of valuable information concerning the circumstances affecting human health, which few writers in this country would have the industry to collect. In this view of the matter, it will not be denied that he has rendered an acceptable service to the profession; and under this impression we could easily look over heresies, or errors of doctrine, touching the wine question and our inestimable Temperance Societies, greater than any we have noted in his agreeable volume. Y.

